# Spatiotemporal trajectory of energy efficiency in the Guangdong-Hong Kong-Macao Greater Bay Area and implications on the route of economic transformation

**DOI:** 10.1371/journal.pone.0307839

**Published:** 2024-09-03

**Authors:** YanFei Lei, Chao Xu, Yunpeng Wang, Xulong Liu, Lili Li, Siyu Chen

**Affiliations:** 1 State Key Laboratory of Organic Geochemistry, Chinese Academy of Sciences, Guangzhou, Guangzhou Institute of Geochemistry, Guangdong, China; 2 University of Chinese Academy of Sciences, Beijing, China; 3 Guangdong Provincial Key Laboratory of Remote Sensing and Geographical Information System, Guangdong Academy of Sciences, Guangzhou Institute of Geography, Guangzhou, Guangdong, China; 4 College of Resources and Environment, Zhongkai University of Agriculture and Engineering, Guangzhou, Guangdong, China; 5 Jinan University-University of Birmingham Joint Institute, Jinan University, Guangzhou, Guangdong, China; Guangzhou Institute of Geography, Guangdong Academy of Sciences, CHINA

## Abstract

The Guangdong-Hong Kong-Macao Greater Bay Area has attracted attention for its extraordinary pace of economic development and is considered to be leading the way in China’s transformation from a manufacturing to an innovation cluster. However, due to rapid economic expansion and rapid urbanization, the Great Bay Area still struggles with low energy efficiency and environmental degradation, which has slowed down the pace of development. Therefore, in order to alleviate energy pressure, promote the country’s sustainable development and gain a competitive advantage in the global market, researching energy efficiency and improving energy utilization efficiency is crucial. In this study, macro-level energy efficiency indicators are constructed using energy consumption data from various cities in the Greater Bay Area for the period from 2000 to 2020, and the spatio-temporal evolution of energy efficiency is analysed. The results show that all cities in the Greater Bay Area experienced an increasing trend in energy efficiency from 2000 to 2019, with significant variation in growth rates and magnitudes between cities. Compared to the nine cities in Guangdong province, Hong Kong and Macao exhibited significantly superior energy efficiency, with Foshan recording the highest growth rate of 14%. In 2020, most cities experienced a decline in energy efficiency due to the COVID-19 pandemic, with Macao experiencing the greatest decrease at 57%. Hong Kong and Macao are both in the "low consumption and high efficiency" target region, while Guangzhou, Shenzhen and Zhuhai are consistently in the "both high" region. Changes in the industrial upgrading index correspond significantly with changes in energy efficiency trajectories, with the transition from primary to secondary and tertiary industries playing a more substantial role. There is no significant association found between the strength of environmental regulation and changes in energy efficiency. The study’s findings indicate that the most effective way to achieve economic transformation in the majority of China’s regions is to combine adequate environmental legislation with industrial structural adjustment.

## Introduction

The Guangdong-Hong Kong-Macao Greater Bay Area is regarded as a crucial spatial carrier for China’s construction of a world-class urban cluster. It plays a vital role in achieving regional coordinated development and participating in global competition [1]. Since the beginning of the reform and opening-up policy, the Greater Bay Area has emerged as a pioneering region in China’s reform efforts, achieving remarkable economic development milestones. It has established world-class industrial clusters and built a comprehensive industry chain system for the incubation of innovative achievements. Currently, the Greater Bay Area is undergoing a transformative phase, transitioning from a manufacturing-oriented bay area to an innovative one [2–6]. However, rapid economic development has also led to issues such as low energy efficiency and ecological environmental degradation. When compared with the three major world-class bay areas of New York, San Francisco, and Tokyo, the Greater Bay Area still lags behind in terms of energy utilization, ecological security, and regional coordination [7–10]. These three bay areas at world level have made rapid progress in the energy transition and show a downward trend in total energy consumption and per capita energy consumption. In contrast, the Greater Bay Area is expected to see a continued increase in energy consumption due to its high economic growth, and the Greater Bay Area’s energy consumption per unit of GDP is still higher than the average of the other three Bay Areas, highlighting its shortcomings in energy efficiency. Research on prospective energy efficiency serves as one of the crucial means for the Greater Bay Area, which is undergoing developmental transformation, to address energy pressures, support national sustainable development, and gain a competitive edge globally.

The Greater Bay Area urban agglomeration possesses distinct characteristics, including ’One Country, Two Systems’, and the integration of front shops and rear factories. There exist significant disparities in the economic development levels among cities in the Greater Bay Area both temporally and spatially. During the period of development and transformation in the Greater Bay Area, different cities exhibit varying speeds and directions of transformation. Summarizing the experiences and lessons learned from cities with fast speeds and accurate directions of development, and forming valuable insights into transformational development paths, can provide beneficial references for other cities. This is a favorable approach for achieving smooth transformation in the Greater Bay Area and promoting regional coordinated development. In such a context, it is worthwhile to delve into energy efficiency, which reflects the quality of energy input and output.

Energy efficiency primarily reflects the contribution of the amount of energy consumed by society to maintaining or promoting economic, social, and environmental sustainable development [11–14]. Liao & Wei and others have summarized seven evaluation indicators for energy efficiency, including macro energy efficiency, physical energy efficiency, thermodynamic energy efficiency, economic energy efficiency, energy factor allocation efficiency, energy factor utilization efficiency, and energy economic efficiency, and pointed out that macro energy efficiency is suitable for medium and long-term research [15–17]. However, it is worth noting that current research on macro-energy efficiency focuses mainly on the national and provincial level. For the urban level, especially for major urban clusters such as the Guangdong-Hong Kong-Macao Greater Bay Area, research on the spatio-temporal differentiation characteristics of macro-energy efficiency and the spatial mechanism of more detailed scale units is still insufficient.

The impact on energy efficiency is influenced by various factors, and the comprehensive review by Yonggang Jin provides valuable insights into this aspect [18]. He points out that several factors interact to influence energy efficiency. These include industrial structure, technological progress, environmental regulations, foreign direct investment (FDI), energy endowment and energy prices, among others. Among these factors, the relationship between industrial structure and environmental regulations and energy efficiency is particularly significant [19–21] (Sun Hao, Lan and Tiantian, 2023; Mu Xianzhong and et al, 2022; Li Baoxin and et al, 2022). In-depth research on these factors helps us to comprehensively understand the dynamic changes in energy efficiency and provides a scientific basis for formulating strategies to improve energy efficiency.

The objective of this study is to conduct an in-depth spatio-temporal development analysis of the macro-energy efficiency of 11 cities in the Guangdong-Hong Kong-Macao Greater Bay Area from 2000 to 2020 using geographic spatial information technology. The specific research objectives are as follows: (1) To reveal the spatiotemporal evolution patterns of macro-energy efficiency in the cities of the Greater Bay Area and apply spatio-temporal trajectory clustering methods to classify each city, identify groups of cities with higher and lower energy utilization efficiency, and analyze the similarities and differences between these groups; (2) Exploring the driving factors influencing energy efficiency changes in Greater Bay Area cities analyzing the impact of industrial structure and environmental regulations on energy efficiency; (3) Providing summaries of city experiences and transformation insights that provide important guidance and references for other cities on the path to energy transition and sustainable development.

## Study area and method

### Study area

The Guangdong-Hong Kong-Macao Greater Bay Area is located in the southeastern part of China, encompassing two special administrative districts of Hong Kong and Macao, as well as nine prefectures in Guangdong Province: Guangzhou, Shenzhen, Zhuhai, Foshan, Huizhou, Dongguan, Zhongshan, Jiangmen, and Zhaoqing. With a total area of 56,000 square kilometers, it is one of the most open and economically vibrant regions in China. Situated in the southern bay of China, it is a typical region characterized by high levels of economic openness and energy input.

Energy consumption in the Guangdong-Hong Kong-Macao Greater Bay Area mainly includes coal, oil, natural gas and renewable energy. In the past ten years, the structure of energy consumption in the Greater Bay Area has been continuously moving towards clean and low-carbon. According to statistics, the proportion of clean energy in the Greater Bay Area’s electricity supply in 2020 was more than 60, and the proportion of electricity and other non-fossil energy sources increased to 26.3%, indicating a positive change in the energy consumption structure.

### Method

#### Construction of energy efficiency model

In this study, the total energy consumption data for the 11 cities in the Greater Bay Area are obtained through inverse modeling based on lighting data, population data, and impervious surface spatial data [15, 22]. The macro energy efficiency indicator *e* (ten thousand yuan/ton of standard coal) was constructed, and the calculation formula is shown in Eq (1), where G represents the regional GDP in ten thousand yuan, and E represents the regional energy consumption in tons of standard coal.


$$e=\frac{G}{E}$$ (1)

The significance of changes in energy efficiency can be tested using the Mann-Kendall significance test. The main advantage is that it is a popular trend test that does not require the data to have a normal distribution. The applicable calculation formula is as follows:

$$Z=\left\{\begin{array}{c} \frac{S-1}{\sqrt{n(n-1)(2n+5)/18}},S>0\\ 0,S=0\\ \frac{S+1}{\sqrt{n(n-1)(2n+5)/18}},S<0 \end{array}\right\}$$ (2)

$$S=\sum_{k=1}^{n-1}\sum_{j=k+1}^{n}sgn(x_{j}-x_{k})$$ (3)

$$sgn(x_{j}-x_{k})=\left\{\begin{array}{c} 1,(x_{j}-x_{k})>0\\ 0,(x_{j}-x_{k})=0\\ -1,(x_{j}-x_{k})<0 \end{array}\right\}$$ (4)

Where *S* is the Mann-Kendall test statistics, *x*_*j*_ and *x*_*k*_ are the energy efficiency values at the time of *j* and *k*. *n* is the time length of the energy efficiency values. The threshold of significance level is ±1.96 (95% significance test).

### Spatiotemporal trajectory analysis

**Fig 1:**
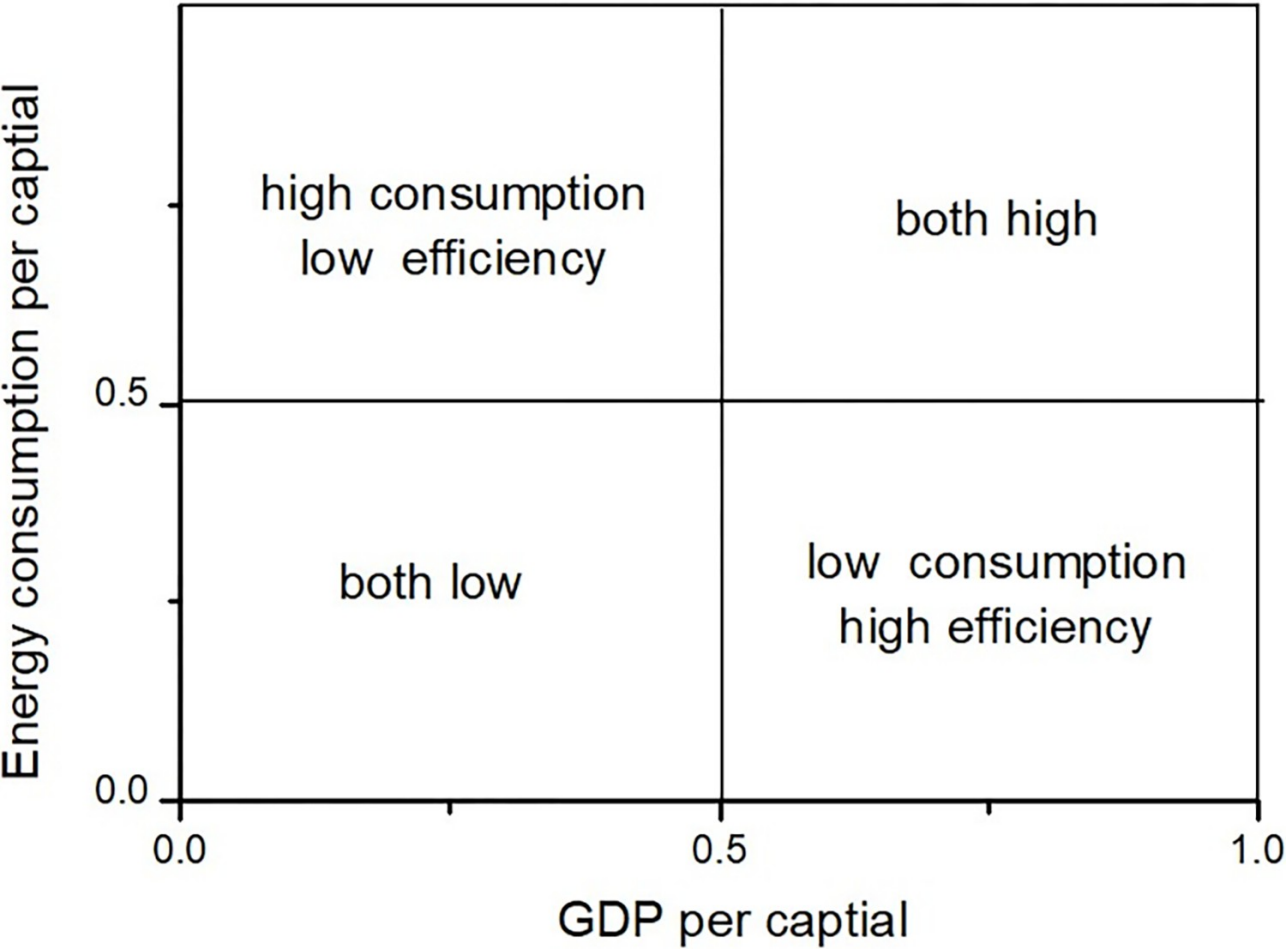
Quadrant chart of energy consumption efficiency.

Spatial-temporal trajectory data is essentially a recorded sequence of the positions and times of moving objects [23]. In practical applications and research, it is generally expressed discretely using sequences of recorded points of position and time. This expression is similar to the representation of annual sequences of energy efficiency for each city. Therefore, introducing methods for mining and analyzing spatial-temporal trajectory data in geographic information science facilitates the measurement and analysis of energy efficiency trajectories. By plotting per capita GDP on the horizontal axis and per capita energy consumption on the vertical axis, a comparative quadrant diagram of energy productivity from 2000 to 2020 can clearly and intuitively display the development trajectory of energy productivity in a region. As shown in Fig 1, the first quadrant represents the "both high" region with high per capita energy consumption and high per capita GDP; the second quadrant represents the "high consumption and low efficiency" region with high per capita energy consumption and low per capita GDP; the third quadrant represents the "both low" region with low per capita energy consumption and low per capita GDP; and the fourth quadrant represents the "low consumption and high efficiency" region with low per capita energy consumption and high per capita GDP, which is the target area for energy productivity development. Typically, a region or city may develop along a trajectory or path from the third quadrant to the second quadrant, and then to the first and fourth quadrants. However, in rapidly developing scenarios, different development trajectories may also be exhibited.

### DTW clustering

The significance of spatial-temporal trajectory data lies in comparing different trajectories to extract similar or dissimilar characteristics [8]. Spatial-temporal trajectory clustering analysis methods are used to distinguish objects with similar and dissimilar behaviors, and measure the similarity and dissimilarity of spatial-temporal trajectories through clustering methods to extract information [24, 25]. Therefore, spatial-temporal trajectory clustering analysis is an important method for studying using spatial-temporal trajectory data.

This study will adopt the method of Dynamic Time Warping (DTW) combined with K-means to cluster the spatial-temporal trajectories of energy productivity in the 11 cities of the Greater Bay Area from 2000 to 2020. DTW is a method for comparing the similarity between two time series. It can handle time series data with different lengths, speeds, and a certain degree of offset. By finding the best matching path between two time series to minimize the total distance between them, it compares their similarity [26, 27]. K-means clustering is a commonly used distance-based clustering algorithm that aims to divide the dataset into K groups so that the distance between samples within the group is as small as possible, while the distance between groups is as large as possible [28–30]. The DTW combined with K-means clustering method first calculates the distance between data points of time series using DTW, and then uses the K-means clustering algorithm to divide the dataset into K groups.

### Advanced industrial structure index

The industrial structure consists of the primary industry, the secondary industry, and the tertiary industry. The process of the shift in emphasis from the primary industry to the secondary and tertiary industries can be regarded as the development of the industrial structure from a low level to a high level. This study utilizes the index of industrial structure sophistication as an indicator of changes in industrial structure. Zheng & Chen constructed the index of industrial structure sophistication (IH) based on the characteristic that the angle between the industry proportion vector and the corresponding coordinate axis changes with the change in industry proportion, quantitatively describing the process of industrial structure sophistication [31]. The formula for calculation is provided in Eqs 5–7, where θ_1_ and θ_2_ respectively measure the degree of transfer from the primary industry to the secondary and tertiary industries and from the secondary industry to the tertiary industry. A higher value indicates a higher degree of transfer, corresponding to a larger index of industrial structure sophistication (IH) and a higher level of industrial structure sophistication. Here, *μ*_1_ and *μ*_2_ represent the angles between the vector (*x*_1_, *x*_2_, *x*_3_) and the vectors(0,1,0)and(0,0,1)respectively​, and σ_1_ is the angle between vectors (*x*_2_, *x*_3_) and(0,1).


$$IH=\theta_{1}+\theta_{2}$$ (5)

$$\theta_{1}=\pi -\mu_{1}-\mu_{2}$$ (6)

$$\theta_{2}=\pi /2-\sigma_{1}$$ (7)

The formula for calculating the angle between vectors is provided in Eq 8, where *x*_1_, *x*_2_,and *x*_3_ respectively represent the proportions of value added of the three industries to GDP, and *n* takes the values of 2 and 3.


$$\theta =a\mathrm{rc}\cos\left[\frac{\sum_{i=1}^{n}\left(x_{i}x_{i,0}\right)}{{(\sum_{i=1}^{n}x_{i}^{2})}^{\frac{1}{2}}{(\sum_{i=1}^{n}x_{i,0}^{2})}^{\frac{1}{2}}}\right]$$ (8)

### Measurement of environmental regulations

The measurement of environmental regulations currently lacks a unified standard. Generally, it is assessed through two approaches. One involves measuring government investments in pollution control, the number and strength of relevant policies enacted by the government, etc. Higher investments and more stringent policies imply stronger environmental regulations. The other approach involves measuring the level of pollutant emissions, where higher emission levels signify weaker environmental regulations. Drawing on the research of Liao et al [32], this study synthesizes environmental pollution indices using emission concentration data to construct the Environmental Regulation Index *ER*_*i*,*t*_, *where higher values of ER*_*i*,*t*_ indicate stronger environmental regulation. The selection of pollutants is primarily based on remote sensing data and environmental reports, including sulfur dioxide, nitrogen dioxide, and PM_2.5_ in the Greater Bay Area.

The specific calculation formula is as follows, where *e*_*i*,*t*,*m*_ represents the emissions of the mth pollutant in city *i* during period *t*, y_i,t_ represents the GDP of city *i* during period *t*.


$${ER}_{i,t}=1/\frac{1}{3}\sum_{m=1}^{3}\frac{{e_{i,t,m}/y}_{i,t}}{\sum_{i=1}^{11}{e_{i,t,m}/y}_{i,t}}$$ (9)

## Results

### Energy efficiency analysis

**Fig 2:**
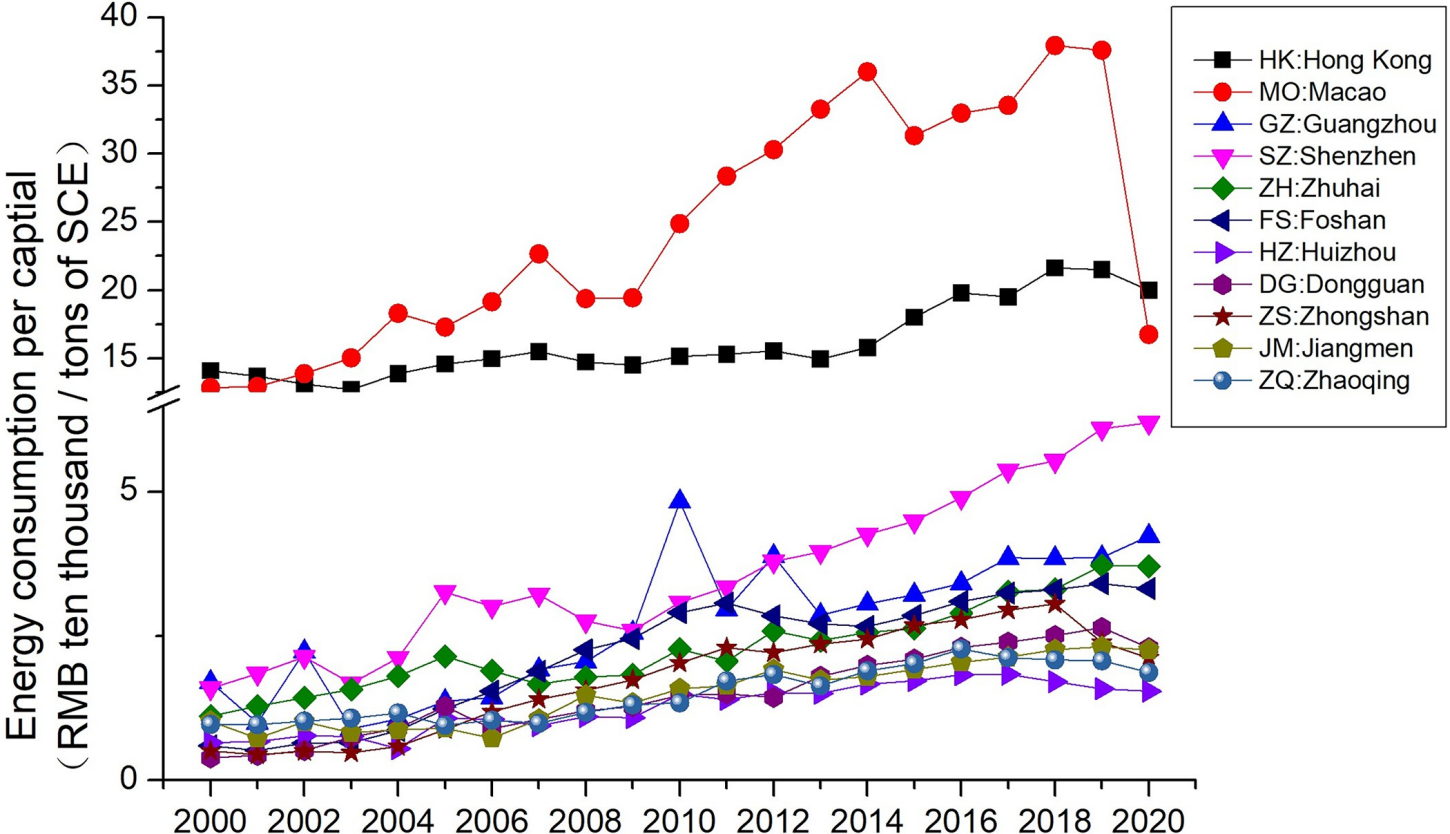
Energy efficiency variation across cities in the Greater Bay Area.

Fig 2 depicts the changes in energy efficiency across cities in the Greater Bay Area from 2000 to 2020. The Mann-Kendall trend test was conducted on the changes in energy efficiency of various cities from 2000 to 2019, excluding the potential impact of the COVID-19 pandemic in 2020 on energy efficiency. The test results indicate that all cities exhibited a significant upward trend in energy efficiency at a 95% confidence level.

From the Fig 2, it can be observed that all cities in the Greater Bay Area showed an upward trend in energy efficiency from 2000 to 2019. However, due to the impact of the COVID-19 pandemic in 2020, most cities experienced a decline in energy efficiency. Among the cities in the Greater Bay Area, Macao and Hong Kong exhibit significantly higher energy efficiency compared to the nine cities in the Pearl River Delta. Macao, in particular, maintained a much higher energy efficiency than Hong Kong from 2003 to 2019, experiencing a rapid increase from 2009 to 2014 with an average annual growth rate of 12.2%. The significant drop in Macao’s GDP in 2020 led to a noticeable decline in energy efficiency.

Among the nine cities in Guangdong Province, Shenzhen holds a leading position in energy efficiency and has shown a unilateral upward trend from 2009 to 2020 with an average annual growth rate of 15.34%. In 2020, Shenzhen’s energy efficiency was 47.62% higher than that of Guangzhou. Guangzhou exhibits a moderate upward trend in energy efficiency and mostly ranks fourth in the Greater Bay Area. Zhuhai showed slight growth in energy efficiency from 2000 to 2005, followed by a decline, falling behind Foshan from 2006 to 2012. However, from 2013 to 2020, Zhuhai’s energy efficiency showed a significant upward trend, surpassing Foshan since 2019 and ranking fifth among the cities in the Greater Bay Area.

Foshan’s energy efficiency displayed a substantial growth trend after 2006, reaching its peak in 2011 with an average annual growth rate of 16.2%. Although Foshan initially surpassed Zhuhai, it was later overtaken again by Zhuhai. As of 2020, Foshan ranks sixth in energy efficiency among the Greater Bay Area cities. Over the period from 2000 to 2020, Foshan had the highest growth rate in energy efficiency at 14%.

Zhongshan initially lagged behind other Greater Bay Area cities from 2000 to 2004 but entered a phase of rapid growth starting in 2005. From 2011 to 2018, Zhongshan’s energy efficiency closely matched that of Foshan and Zhuhai. With an average annual growth rate of 11.6% from 2005 to 2018, Zhongshan was ranked seventh in energy efficiency until 2018, but it declined significantly after 2018, dropping to the ninth position in 2020.

Dongguan, starting from the bottom in 2000 and 2001, gradually improved its energy efficiency and consistently ranked eighth in the Greater Bay Area from 2007 to 2018. Since 2019, Dongguan has been in the seventh position, and over the 21-year period from 2000 to 2020, it achieved an average annual growth rate of 11.6%.

Jiangmen maintained the ninth position in energy efficiency from 2004 to 2019. The period from 2006 to 2012 marked a rapid growth phase for Jiangmen, with an average annual growth rate of 17.59%. However, its energy efficiency started declining after 2018.

Zhaoqing witnessed a significant decline in its ranking in energy efficiency among Greater Bay Area cities. From 2000 to 2004, it held the sixth position but later fluctuated between the ninth and tenth positions. After 2017, it consistently ranked tenth in the Greater Bay Area.

Huizhou consistently ranked low in energy efficiency among Greater Bay Area cities. Except for a slightly higher ranking than Zhaoqing in 2005 and 2006, Huizhou’s energy efficiency remained at the bottom in all other years. From 2018 to 2020, its energy efficiency was only half of Foshan’s, indicating a significant gap.

### The cluster analysis of energy efficiency development trajectories

**Fig 3:**
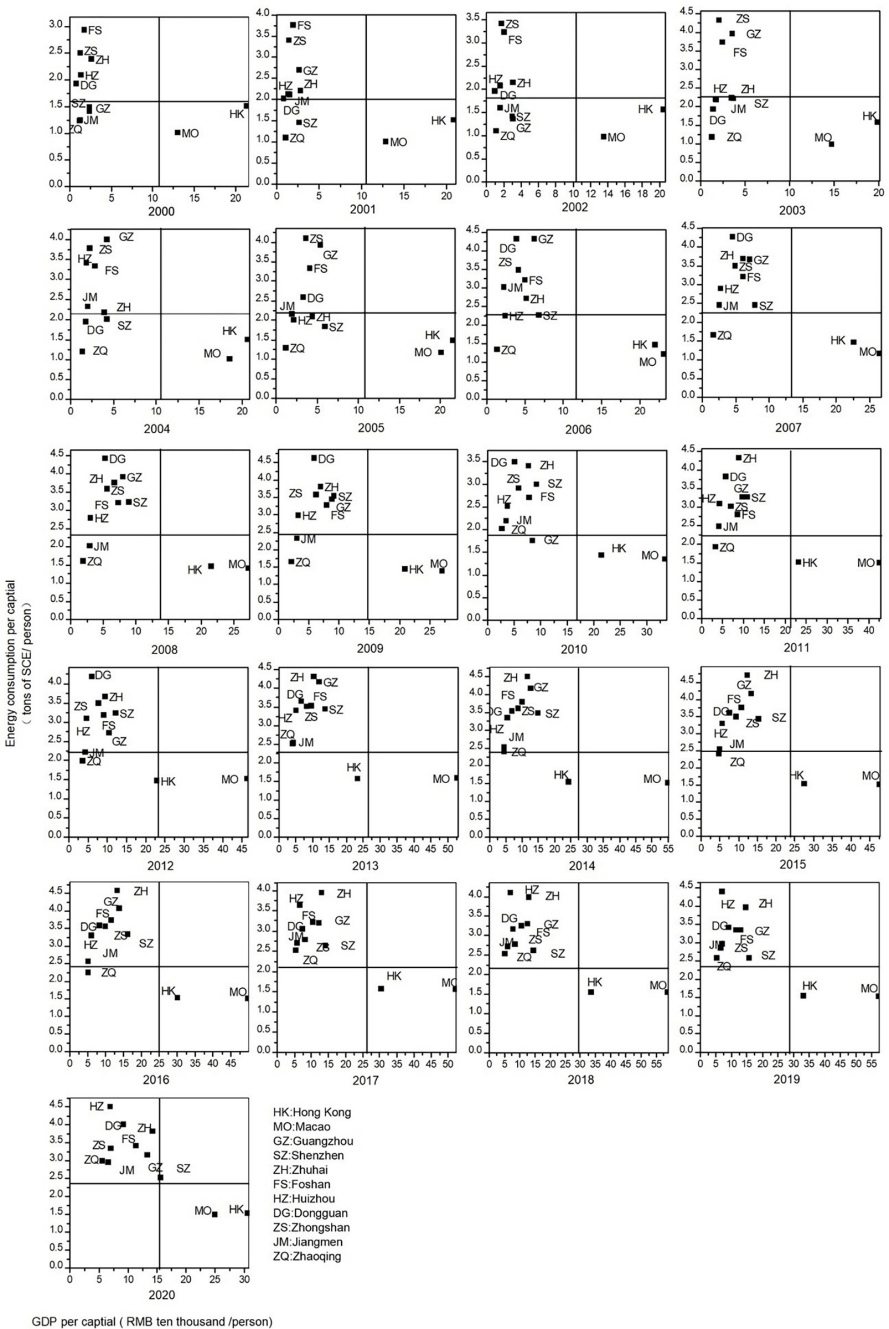
Changes in GDP and energy consumption per capital of each city.

Based on the construction of the macro energy efficiency indicators, a comparative quadrant chart of per capital GDP and per capital energy consumption for the 11 cities in the Greater Bay Area was plotted, as shown in Fig 3. It can be clearly observed that the energy efficiency of each city exhibits diverse developmental trajectories over time and space.

Fig 3 illustrates the variation in per capital energy consumption and per capital GDP for the 11 cities in the Greater Bay Area. It also represents the changes in the development trajectory of energy efficiency on the quadrant chart. It can be observed that, except for the period of 2012–2014 when Hong Kong was at the edge of the third-fourth quadrant, Hong Kong consistently remains in the "low consumption and high efficiency" fourth quadrant for the rest of the time, along with Macao throughout the study time series. Shenzhen exhibits a trajectory transitioning from the "both low" third quadrant through the "high consumption and low efficiency" second quadrant to the "both high" first quadrant. In the year 2000, many cities in the Pearl River Delta, among the 9 cities, were still in the "both low" region, but by 2017, all had entered the "high consumption and low efficiency" region.

By applying DTW combined with K-means clustering to the spatiotemporal trajectory data of energy efficiency development in the 11 cities of the Greater Bay Area, Hong Kong and Macao are each grouped separately, while the 9 cities in the Pearl River Delta are clustered into two categories. To gain a clearer and more intuitive understanding of the distances between Hong Kong, Macao, and other cities, this study calculated the Euclidean distances of the spatiotemporal trajectories between Hong Kong, Macao, and the 9 cities in the Pearl River Delta. The Euclidean distance of the trajectory between Hong Kong and Macao is 7.87, indicating a relatively close trajectory. However, the trajectories of Hong Kong and Macao differ significantly from those of other cities, all exceeding 10. Among them, the trajectories of Shenzhen and Guangzhou are relatively close to those of Hong Kong and Macao compared to the other 7 cities (Hong Kong to Shenzhen is 12.40, Hong Kong to Guangzhou is 14.70, Macao to Shenzhen is 15.14, and Macao to Guangzhou is 17.60).

**Fig 4:**
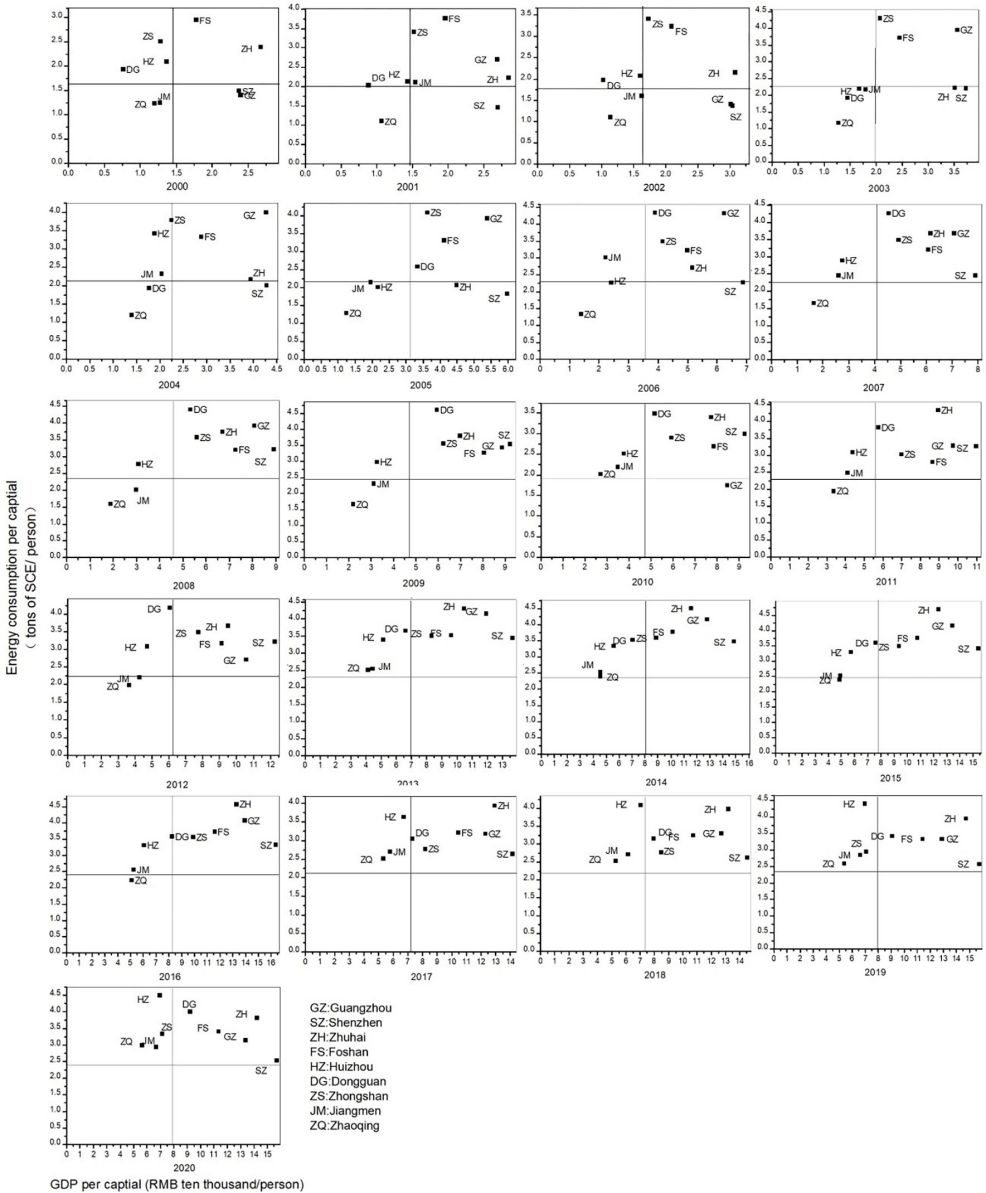
Changes in GDP and energy consumption per capital of each city in the Pearl River Delta.

In comparison to Fig 3, it can be observed that the clustering results do not align perfectly with the level of energy efficiency. A more in-depth analysis is required for the clustering outcomes. Due to the substantial differences between Hong Kong and Macao compared to other cities, the study excludes Hong Kong and Macao. Subsequently, a new comparative quadrant chart of per capital GDP and per capital energy consumption for the remaining 9 cities in the Pearl River Delta of the Greater Bay Area is plotted, as shown in Fig 4.

**Fig 5:**
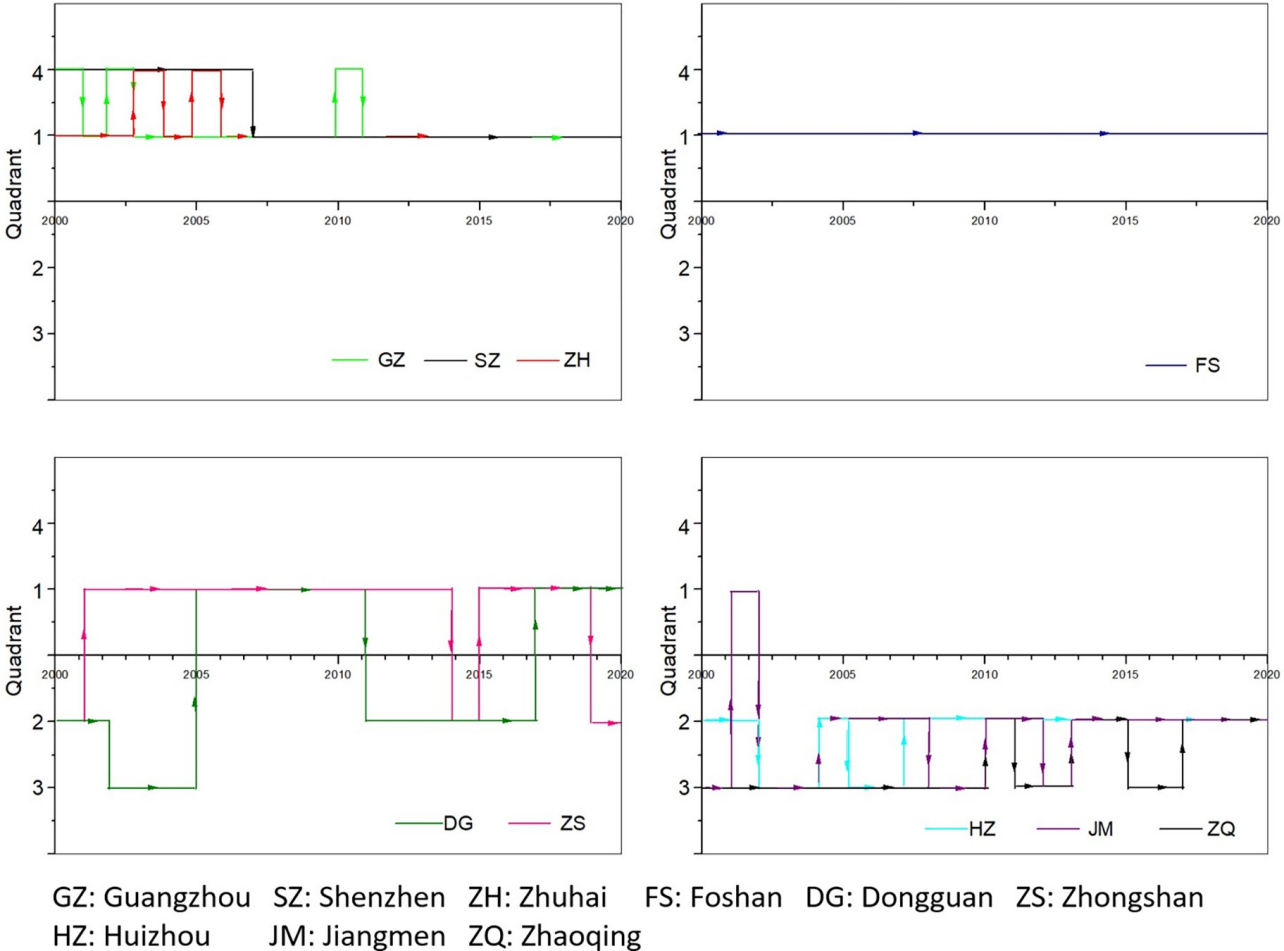
Quadrant chart energy efficiency development trajectory of the four groups.

Fig 4 illustrates the energy efficiency changes in nine cities, revealing certain differences among them. Similarly, utilizing the DTW combined with K-means clustering method, an analysis of the energy efficiency trajectories of the nine cities in the Pearl River Delta region of the Greater Bay Area was conducted. The clustering results for the nine cities in the Pearl River Delta region can be categorized into four groups: the first group includes Guangzhou, Shenzhen, and Zhuhai; the second group consists of Foshan; the third group comprises Dongguan and Zhongshan; and the fourth group includes Huizhou, Jiangmen, and Zhaoqing. Comparing the energy efficiency clustering results leads to the findings presented in Fig 5.

Cluster 1 of the clustering results comprises Guangzhou, Shenzhen, and Zhuhai. The spatiotemporal trajectory similarity feature is that most of the time is spent in the "both high" quadrant. Shenzhen moved from the "low consumption and high efficiency" quadrant in 2007 to the "both high" quadrant, indicating that, like other cities in the Pearl River Delta in the Greater Bay Area, Shenzhen entered a high-energy consumption area. Guangzhou and Zhuhai exhibit similar trajectories, oscillating between the "low consumption and high efficiency" fourth quadrant and the "both high" first quadrant. Their movements towards high-energy consumption areas are particularly significant, especially Zhuhai, whose per capital energy consumption ranked first among the nine cities in the Pearl River Delta in most years from 2011 to 2017 (except for 2012 when it ranked second).

Cluster 2 of the clustering results corresponds to Foshan, with the energy efficiency spatiotemporal trajectory consistently located in the "both high" quadrant. However, when combined with Fig 4, it is evident that Foshan’s per capital energy consumption shows a declining trend, indicating a movement from the "both high" area towards the goal of "low consumption and high efficiency."

Cluster 3 of the clustering results includes Dongguan and Zhongshan, with similar spatiotemporal trajectory features, spending most of the time fluctuating between the "high consumption and low efficiency" second quadrant and the "both high" first quadrant. Dongguan briefly stayed in the "both low" area from 2002 to 2005, leaping directly from the "both low" area to the "both high" area in 2005. Zhongshan, on the other hand, started descending from the "both high" area to the "high consumption and low efficiency" area in 2019.

Cluster 4 of the clustering results involves Huizhou, Jiangmen, and Zhaoqing, with similar spatiotemporal trajectory features spending most of the time changing between the "both low" area and the "high consumption and low efficiency" area. Huizhou shifted from "high consumption and low efficiency" in 2002 to the "both low" area and, after some fluctuations, returned to and remained in the "high consumption and low efficiency" area from 2007 onwards. Jiangmen started from the "both low" area, briefly stayed in the "both high" area from 2001 to 2002, returned to the "both low" area in 2002, and subsequently fluctuated between the "both low" and "high consumption and low efficiency" areas. Jiangmen stayed in the "high consumption and low efficiency" area from 2013. Zhaoqing mostly stayed in the "both low" area, with brief stays in the "high consumption and low efficiency" area in 2010–2011 and 2013–2015, until moving from the "both low" to the "high consumption and low efficiency" area in 2017.

Combining Fig 3, Fig 4, and Fig 5, it is evident that the energy efficiency development trajectories of the 11 cities in the Greater Bay Area vary, but within each category, there are similar characteristics. This reflects the similarity in development patterns among the cities in the Pearl River Delta, though not entirely identical. The mechanisms leading to these results are not yet clearly understood. This study will integrate the Advanced Industrial Structure Index and environmental regulation measures to explore the driving influences behind the spatiotemporal trajectory changes in energy efficiency.

### Analysis of Driving Forces of Energy Efficiency Development Trajectories

#### Calculation and analysis of the advanced industrial structure index

**Fig 6:**
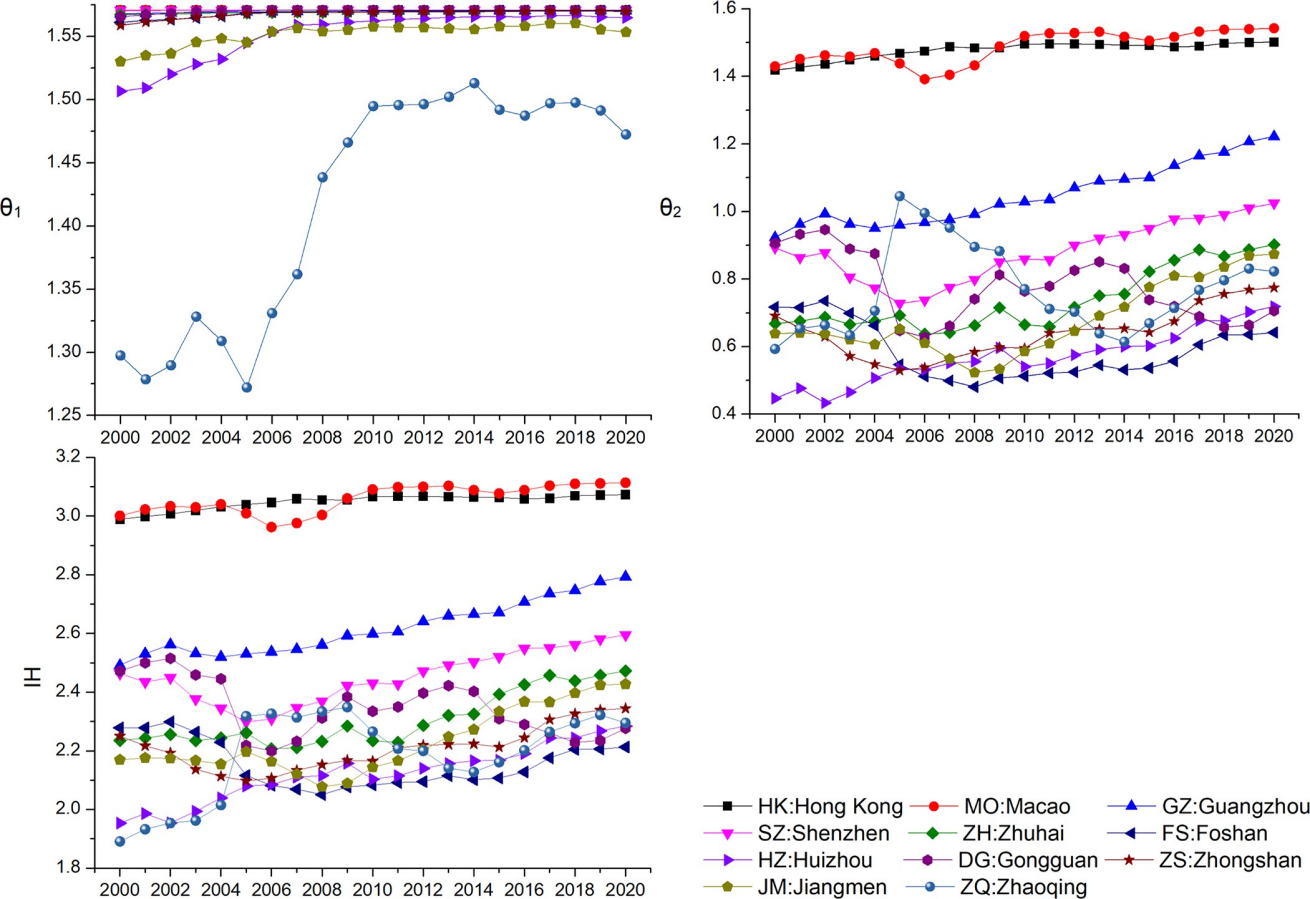
The change of advanced industrial structure index of each city.

According to Eqs 5–8, the Advanced Industrial Structure Index for each city in the Greater Bay Area was calculated over the years, and the changes are illustrated in Fig 6.

Comparing Fig 2 and Fig 6, it can be observed that although there is no direct correspondence between the energy efficiency and the Advanced Industrial Structure Index among cities, there is a significant correlation in the clustering results of the spatiotemporal trajectory of energy efficiency. Macao and Hong Kong exhibit considerably higher levels of both energy efficiency and Advanced Industrial Structure Index than the other 9 cities in the Greater Bay Area. Guangzhou and Shenzhen consistently rank high in the Advanced Industrial Structure Index among the 9 cities, with Zhuhai occupying the third position after 2014. Foshan’s Advanced Industrial Structure Index has a distinct trend compared to other cities, experiencing a rapid decline from 2000 to 2007 and consistently ranking at the bottom among the Greater Bay Area cities. Dongguan’s Advanced Industrial Structure Index was among the top cities in the Greater Bay Area from 2000 to 2004, started a declining trend from 2002, experienced a significant rise in 2005, remained stable from 2009 to 2014, and then entered another declining trend. Zhongshan’s Advanced Industrial Structure Index trend from 2000 to 2015 is similar to Dongguan’s, with a slight upward trend from 2016 to 2020. Jiangmen, Huizhou, and Zhaoqing initially ranked among the bottom three in the Advanced Industrial Structure Index in the Greater Bay Area. Over time, they all showed an overall upward trend. Among them, Zhaoqing had the largest fluctuations, consistently ranking at the bottom from 2000 to 2004, experiencing a significant leap in growth from 2005 to 2009, followed by a pronounced decline from 2009 to 2014. After 2015, Zhaoqing demonstrated a slow upward trend.

Subdividing the Advanced Industrial Structure Index in the Greater Bay Area reveals that the transition from the primary industry to the secondary and tertiary industries contributes significantly to the overall Advanced Industrial Structure Index of the cities. From Fig 4, it is evident that the growth rate of the transition from the primary industry to the secondary and tertiary industries in Zhaoqing, Huizhou, and Jiangmen is higher than that of other cities. Although Hong Kong, Macao, and Shenzhen rank high in the transition index, their growth is almost negligible. Guangzhou experienced an accelerated transition from the primary industry to the secondary and tertiary industries from 2000 to 2010, while Foshan showed an accelerated transition from the primary industry to the secondary and tertiary industries from 2000 to 2007. From 2000 to 2020, among the 11 cities in the Greater Bay Area, except for Foshan and Dongguan, the transition from the secondary industry to the tertiary industry showed a clear upward trend.

Foshan exhibited a significant decline for most of the time, with slow growth from 2015 to 2020, indicating a consistent emphasis on the dominance of the secondary industry. Dongguan experienced significant fluctuations in the transition from the secondary industry to the tertiary industry, reflecting frequent changes in the development direction of Dongguan’s industrial structure.

Through a comprehensive analysis of the Advanced Industrial Structure Index, it is evident that changes in industrial structure play a significant role as one of the main factors driving the spatiotemporal trajectories of energy efficiency in cities across the Greater Bay Area.

#### Calculation and analysis of environmental regulation intensity

**Fig 7:**
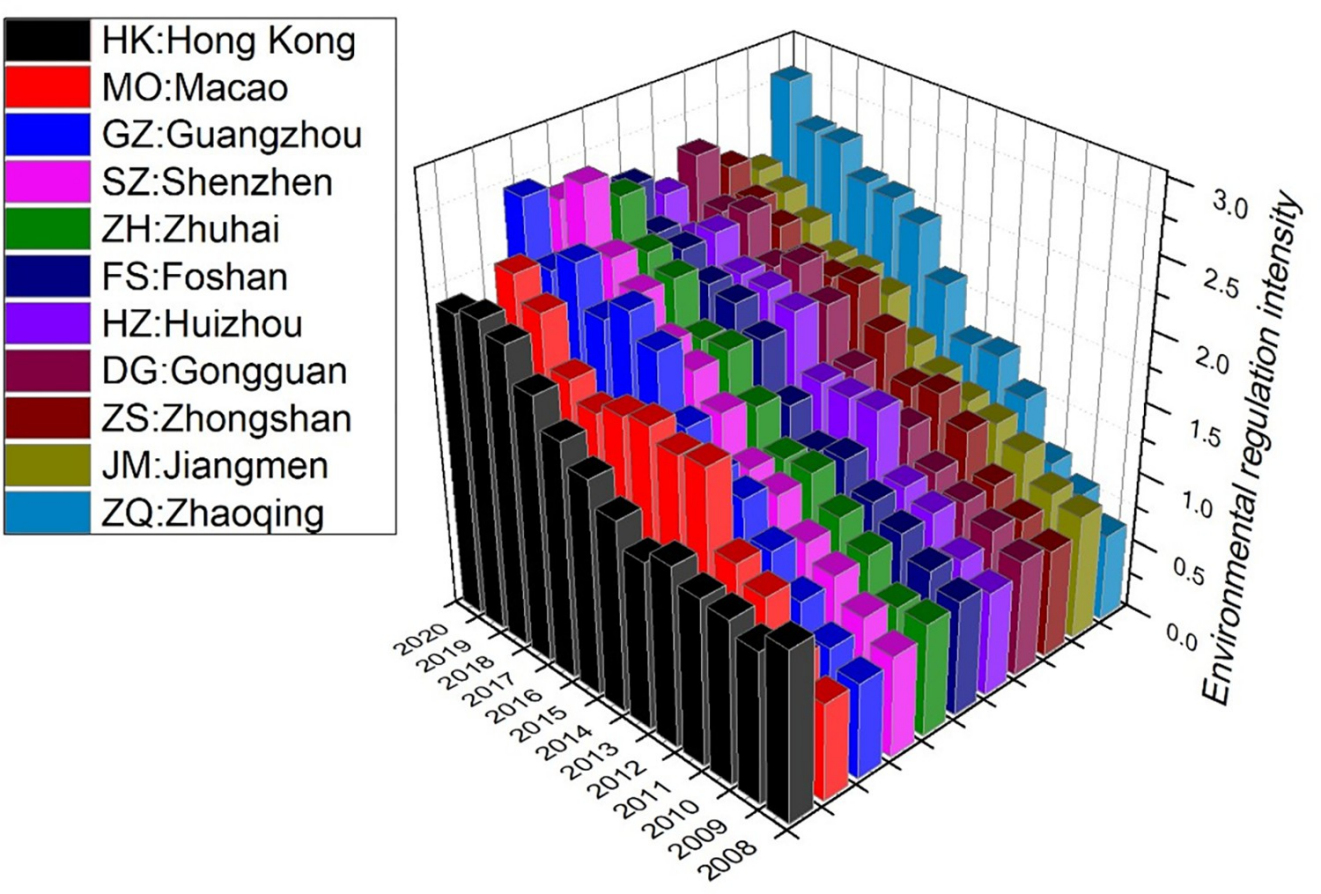
Environmental regulation intensity of each city.

Due to data collection constraints, this study computed the environmental regulation intensity for each city in the Greater Bay Area from 2008 to 2020 using Eq 9, as depicted in Fig 7.

From the Fig 7, it is evident that the environmental regulation intensity of most cities is on an upward trend. Among them, Zhaoqing has experienced the most significant increase, rising from the bottom in the Greater Bay Area in 2008 to the top in 2020. Hong Kong and Macao show relatively modest increases in environmental regulation intensity. However, both Hong Kong and Macao maintained high levels of environmental regulation intensity at the beginning of the time series. From 2008 to 2010, Hong Kong ranked first in environmental regulation intensity in the Greater Bay Area, while Macao ranked second. From 2012 to 2014, Macao occupied the top position in the Greater Bay Area but was later replaced by cities in the Pearl River Delta. After 2014, both Hong Kong and Macao gradually fell behind other cities in the Greater Bay Area in terms of environmental regulation intensity.

Guangzhou, Shenzhen, Zhuhai, and Foshan exhibit a consistent growth pattern in environmental regulation intensity, with rapid growth starting in 2015. Huizhou and Dongguan show similar trends in environmental regulation intensity, both entering a phase of rapid growth from 2012. Zhongshan and Jiangmen, on the other hand, experienced significant growth starting in 2011 but entered a slow growth phase around 2016, with their environmental regulation intensity lagging behind Huizhou and Dongguan.

In summary, the environmental regulation intensity in the Greater Bay Area exhibits contrasting features in different time periods: from 2008 to 2014, cities with high environmental regulation intensity tended to have relatively higher energy efficiency. However, from 2014 to 2020, a reverse relationship is observed, where cities with high environmental regulation intensity have a higher proportion of lower energy efficiency.

By comparing Fig 2 and Fig 7, it is evident that the changes in environmental regulation intensity do not exhibit a clear and significant correlation with the spatiotemporal trajectory changes in energy efficiency. The impact mechanism of environmental regulation intensity on energy efficiency appears to be more complex and does not demonstrate a pronounced driving effect, unlike the clear influence seen in the changes in industrial structure.

## Discussion

In the development of an economy in a region, there is traditionally an evolution trajectory from the third quadrant to the second quadrant, then to the first quadrant and finally to the fourth quadrant. This trajectory development can be observed even in highly developed regions such as the Guangdong-Hong Kong-Macao Greater Bay Area. Hong Kong and Macao made the transition in their early economic development phase and have consistently remained in the fourth quadrant, the region characterized by low energy consumption and high economic performance. In contrast, the development trajectories of the other nine cities in the Pearl River Delta are essentially similar, differing only in the order of progression.

Overall, the adjustment of industrial structure emerges as the primary driving factor for changes in energy efficiency in each city. The transition from the first industry to the second industry inevitably leads to rapid economic development, but at the same time to a significant decline in energy efficiency. The transition from the second industry to the third industry can be regarded as a necessary way to improve energy efficiency. The development path of Shenzhen basically confirms this transition. Although environmental regulation is an important measure to maintain regional environmental quality, environmental regulation alone cannot significantly improve energy efficiency. They need to be combined with industrial adjustments. This observation provides valuable insights for the economic transition of most cities in the Pearl River Delta and other regions or cities across China.

This study also has certain limitations. Although the study recognized that the structure of energy consumption is a key factor, it did not comprehensively analyze the proportion of different types of energy (such as coal, oil, natural gas, renewable energy, etc.) in total energy consumption and their specific impacts on energy consumption and energy efficiency because it was difficult to obtain data on energy structure. This limits to some extent a comprehensive understanding of the issues related to energy consumption and energy efficiency.

## Conclusion

Using macro energy efficiency indicators, the study examines how energy efficiency has changed in 11 cities in the Greater Bay Area between 2000 and 2020. It establishes indicators for the strength of environmental regulations and the progress of industrial structures, and then examines how these factors relate to energy efficiency. The main conclusions are as follows:

1. From 2000 to 2019, energy efficiency increased in all Greater Bay Area cities. However, the COVID-19 pandemic led to a decline in efficiency in 2020. Hong Kong and Macao showed significantly higher efficiency compared to mainland cities. Cities such as Shenzhen, Zhuhai and the Guangzhou-Foshan region, which is adjacent to Hong Kong and Macao, were identified as highly efficient areas, while Zhaoqing and Huizhou showed lower efficiency.
2. In 2000, most cities in the Greater Bay Area fell into the "low consumption and low efficiency" region on a quadrant chart plotting GDP per capita against energy consumption per capita. This suggested they were at an early stage of development compared to Hong Kong and Macao, which were in the "low consumption and high efficiency" region. However, by 2017, all cities transitioned to the "high consumption and low efficiency" region, indicating a trend towards a coarse high-energy model.
3. Spatio-temporal trajectory data were clustered through an in-depth study of nine cities in the Pearl River Delta. The trajectories were limited to the "high consumption and high efficiency" and "low consumption and high efficiency" zones, with Guangzhou, Shenzhen and Zhuhai mainly concentrated in this region. Despite a downward trend in per capita energy consumption, Foshan remained continuously in the "high consumption and high efficiency" zone. The "high consumption and low efficiency" and "high consumption and high efficiency" quadrants fluctuated between Dongguan and Zhongshan, highlighting the difficulties in achieving high output with low energy consumption. Compared to the other cities, Huizhou, Jiangmen and Zhaoqing appear to be still at an early stage of development, as evidenced by their predominant oscillation between the "low consumption and low efficiency" and "high consumption and low efficiency" zones. To summarize, the eleven cities in the Greater Bay Area have a variety of energy production trajectories, with each group showing certain similarities, indicating a certain degree of common development trends.
4. The energy efficiency trajectory changes significantly match with the clustering results for changes in the industrial sophistication index. One of the most important forces influencing the spatiotemporal trajectory of energy efficiency in the cities of the Greater Bay Area cities is the change in industrial structure. Higher energy efficiency correlates with a higher industrial sophistication index, with the shift from primary to secondary and tertiary industries accounting for a larger portion of the driving force.
5. There are varying trends in the intensity of environmental regulation in the Greater Bay Area over time. In 2008–2014, cities with higher environmental regulation intensity showed comparatively higher energy efficiency. However, between 2014 and 2020, there was an inverse relationship, with cities with higher environmental regulation intensity having a higher proportion of lower energy efficiency. There is no clear evidence of a relationship between changes in the intensity of environmental regulation and fluctuations in the spatiotemporal trajectory of energy efficiency. For the more complex driving mechanism of environmental regulation intensity on energy efficiency, there is no analogous influence to that of industrial structure.

## Supporting information

S1 Table Energy efficiency of each city in the Greater Bay Area from 2000 to 2020. (PDF)

S2 Table Advanced industrial structure index of each city in the Greater Bay Area from 2000 to 2020. (PDF)

S3 Table Environmental regulation intensity of each city in the Greater Bay Area from 2008 to 2020. (PDF)
