# Supplementary material for: Spatiotemporal trajectory of energy efficiency in the Guangdong-Hong Kong-Macao Greater Bay Area and implications on the route of economic transformation
Source: PLoS One. 2024 Sep 3;19(9):e0307839. doi: 10.1371/journal.pone.0307839 (PMC11371227; doi:10.1371/journal.pone.0307839)
Supplement: S1 Table — (PDF) [file pone.0307839.s001.pdf]

Energy Efficiency (ten thousand yuan/ton of standard coal)											
Year	HK	MO	GZ	SZ	ZH	FS	HZ	DG	ZS	JM	ZQ
2000	14.085	12.855	1.705	1.597	1.115	0.604	0.655	0.395	0.514	1.027	0.973
2001	13.686	12.932	0.999	1.853	1.288	0.522	0.675	0.436	0.447	0.734	0.969
2002	13.115	13.875	2.236	2.140	1.436	0.649	0.774	0.523	0.507	1.020	1.031
2003	12.708	15.040	0.899	1.681	1.578	0.658	0.764	0.753	0.480	0.829	1.082
2004	13.873	18.302	1.065	2.127	1.811	0.865	0.552	0.905	0.591	0.878	1.167
2005	14.577	17.295	1.374	3.266	2.152	1.237	1.077	1.286	0.882	0.905	0.955
2006	14.975	19.146	1.438	3.016	1.902	1.549	1.066	0.898	1.191	0.735	1.046
2007	15.494	22.655	1.924	3.221	1.670	1.891	0.948	1.061	1.410	1.062	0.993
2008	14.732	19.376	2.061	2.764	1.789	2.266	1.108	1.206	1.564	1.477	1.179
2009	14.505	19.440	2.562	2.596	1.831	2.457	1.087	1.283	1.743	1.342	1.313
2010	15.131	24.876	4.835	3.088	2.278	2.911	1.495	1.478	2.038	1.588	1.348
2011	15.305	28.353	2.969	3.353	2.068	3.075	1.404	1.500	2.304	1.646	1.726
2012	15.545	30.303	3.889	3.797	2.591	2.857	1.521	1.445	2.215	1.920	1.838
2013	14.939	33.267	2.867	3.964	2.428	2.718	1.511	1.807	2.365	1.741	1.641
2014	15.790	36.023	3.065	4.269	2.566	2.671	1.670	1.996	2.451	1.802	1.901
2015	18.015	31.338	3.222	4.495	2.634	2.860	1.728	2.104	2.691	1.924	2.027
2016	19.794	32.982	3.420	4.905	2.900	3.102	1.833	2.306	2.785	2.047	2.280
2017	19.512	33.557	3.861	5.377	3.277	3.249	1.841	2.395	2.957	2.138	2.128
2018	21.626	37.951	3.853	5.543	3.316	3.308	1.712	2.514	3.059	2.270	2.091
2019	21.501	37.608	3.867	6.101	3.727	3.412	1.588	2.652	2.391	2.319	2.076
2020	19.992	16.753	4.236	6.202	3.715	3.326	1.548	2.300	2.127	2.265	1.877
