# Supplementary material for: Spatiotemporal trajectory of energy efficiency in the Guangdong-Hong Kong-Macao Greater Bay Area and implications on the route of economic transformation
Source: PLoS One. 2024 Sep 3;19(9):e0307839. doi: 10.1371/journal.pone.0307839 (PMC11371227; doi:10.1371/journal.pone.0307839)
Supplement: S2 Table — (PDF) [file pone.0307839.s002.pdf]

Advanced Industrial Structure Index											
Year	HK	MO	GZ	SZ	ZH	FS	HZ	DG	ZS	JM	ZQ
2000	2.989	3.001	2.491	2.464	2.235	2.278	1.953	2.472	2.250	2.169	1.891
2001	2.998	3.022	2.531	2.435	2.243	2.278	1.986	2.500	2.217	2.176	1.932
2002	3.007	3.033	2.562	2.449	2.256	2.299	1.954	2.515	2.193	2.174	1.954
2003	3.019	3.030	2.532	2.376	2.234	2.264	1.994	2.459	2.137	2.167	1.962
2004	3.031	3.040	2.520	2.344	2.244	2.229	2.039	2.445	2.113	2.155	2.015
2005	3.039	3.009	2.530	2.298	2.262	2.116	2.080	2.218	2.097	2.197	2.318
2006	3.046	2.962	2.537	2.309	2.207	2.082	2.085	2.199	2.107	2.164	2.327
2007	3.058	2.976	2.546	2.346	2.211	2.069	2.110	2.232	2.133	2.120	2.314
2008	3.055	3.003	2.561	2.369	2.232	2.051	2.116	2.312	2.153	2.077	2.334
2009	3.055	3.060	2.593	2.421	2.284	2.077	2.157	2.384	2.167	2.089	2.349
2010	3.067	3.090	2.599	2.430	2.234	2.083	2.103	2.335	2.165	2.144	2.265
2011	3.067	3.098	2.606	2.428	2.229	2.091	2.114	2.350	2.210	2.166	2.207
2012	3.067	3.099	2.640	2.471	2.286	2.095	2.140	2.397	2.219	2.203	2.199
2013	3.065	3.103	2.661	2.491	2.320	2.115	2.156	2.422	2.222	2.248	2.141
2014	3.064	3.088	2.666	2.503	2.325	2.102	2.166	2.403	2.223	2.273	2.128
2015	3.063	3.077	2.671	2.520	2.392	2.107	2.168	2.309	2.212	2.334	2.161
2016	3.058	3.088	2.707	2.549	2.426	2.128	2.191	2.290	2.245	2.368	2.202
2017	3.060	3.103	2.736	2.551	2.457	2.176	2.244	2.260	2.306	2.366	2.264
2018	3.069	3.110	2.746	2.561	2.438	2.205	2.243	2.229	2.326	2.397	2.294
2019	3.071	3.111	2.777	2.581	2.458	2.206	2.268	2.235	2.339	2.425	2.322
2020	3.073	3.113	2.793	2.595	2.472	2.212	2.284	2.277	2.344	2.427	2.295
