# Supplementary material for: Spatiotemporal trajectory of energy efficiency in the Guangdong-Hong Kong-Macao Greater Bay Area and implications on the route of economic transformation
Source: PLoS One. 2024 Sep 3;19(9):e0307839. doi: 10.1371/journal.pone.0307839 (PMC11371227; doi:10.1371/journal.pone.0307839)
Supplement: S3 Table — (PDF) [file pone.0307839.s003.pdf]

Environmental Regulation Intensity											
**Year**	**HK**	**MO**	**GZ**	**SZ**	**ZH**	**FS**	**HZ**	**DG**	**ZS**	**JM**	**ZQ**
2008	1.21	0.69	0.68	0.72	0.8	0.82	0.74	0.83	0.77	0.89	0.62
2009	1.07	0.86	0.8	0.87	0.8	0.92	0.86	0.94	0.88	0.95	0.81
2010	1.16	1.17	1	1.04	1.05	1.07	1.08	1.03	1.03	1.14	0.94
2011	1.18	1.28	1.23	1.15	1.2	1.18	1.16	1.14	1.31	1.25	1.31
2012	1.28	1.81	1.47	1.37	1.4	1.37	1.59	1.35	1.48	1.31	1.52
2013	1.19	1.78	1.53	1.44	1.39	1.32	1.58	1.37	1.43	1.4	1.51
2014	1.36	1.87	1.74	1.72	1.65	1.54	1.59	1.57	1.72	1.52	1.85
2015	1.53	1.83	2.15	1.91	1.94	1.9	1.99	1.93	1.97	1.79	2.19
2016	1.68	1.75	2.32	2	1.92	2.06	2.05	2.12	1.93	1.87	2.29
2017	1.89	1.88	2.17	2.24	2.24	2.1	2.07	2.02	1.97	1.89	2.32
2018	2.13	2.23	2.46	2.39	2.33	2.25	2.27	2.31	2.12	2.05	2.52
2019	2.2	2.4	2.32	2.79	2.62	2.28	2.14	2.24	2.22	2.19	2.53
2020	2.15	1.69	2.72	2.61	2.53	2.52	2.37	2.55	2.38	2.27	2.81
